# TKT drives renal cell carcinoma progression through metabolic reprogramming and synergistic interaction with PKM2

**DOI:** 10.1038/s41420-025-02837-7

**Published:** 2025-11-18

**Authors:** Qianqing Wang, Anqun Tang, Qingxin Zhuang, Haifeng Xu, Shiping Xu, Jing Zhang, Yao Wang, Liantao Li, Sufang Chu, Yan Wang, Jin Bai, Minle Li, Rui Zhang

**Affiliations:** 1 Xinxiang Central Hospital, The Fourth Clinical College of Xinxiang Medical University, Xinxiang, 453000 Henan China; 2 Cancer Institute, Xuzhou Medical University, Xuzhou, 221004 Jiangsu China; 3 Department of Oncology, People’s Hospital of Ningxia Hui Autonomous Region, Ningxia Medical University, Yinchuan, 750011 Ningxia China; 4 Department of Thoracic Surgery, Xuzhou Medical University Affiliated Hospital Sihong Branch, The First People’s Hospital of Sihong County, Suqian, 223800 Jiangsu China; 5 Department of Pharmacy, Affiliated Hospital of Xuzhou Medical University, Xuzhou, 221000 Jiangsu China; 6 Jiangsu Center for the Collaboration and Innovation of Cancer Biotherapy, Xuzhou Medical University, Xuzhou, 221004 Jiangsu China; 7 Department of Respiratory and Critical Care Medicine, Foshan Fosun Chancheng Hospital, Foshan, 528031 Guangdong China

**Keywords:** Urological cancer, Cancer metabolism

## Abstract

Renal cell carcinoma (RCC) undergoes profound metabolic reprogramming to fuel its aggressive progression and metastatic dissemination. While transketolase (TKT), a central metabolic enzyme, has been shown to exert dichotomous roles as either oncogenic or tumor-suppressive factors across different malignancies, its functional significance in RCC pathogenesis remains inadequately defined. In this study, we demonstrate that TKT promotes glucose metabolism in RCC by enhancing glycolysis, thereby supporting tumor progression. TKT expression is significantly elevated in RCC tissues and correlates with poor patient prognosis. Mechanistically, we uncovered a novel functional axis between TKT and the glycolytic gatekeeper pyruvate kinase M2 (PKM2), where their coordinated action drives metastatic progression and metabolic adaptation in RCC. Knockdown of PKM2 significantly impaired the TKT-mediated increases in glycolysis, cell proliferation, and invasive potential. Taken together, our findings highlight TKT as a pivotal regulator of metabolic reprogramming in RCC and suggest its potential as a therapeutic target for the treatment of this malignancy.

## Introduction

Renal cell carcinoma (RCC), derived mainly from renal tubular epithelial cells, comprises ~90% of kidney malignancies, with clear cell RCC (ccRCC) as the dominant subtype (70–80%) [1, 2]. Other subtypes include papillary, chromophobe RCC, and rare variants. Its asymptomatic early stage and resistance to conventional therapies remain major clinical challenges [3, 4].

A defining feature of RCC lies in its profound metabolic reprogramming, which distinguishes it from normal renal parenchyma and fuels its pathogenic progression [5]. This metabolic rewiring is closely intertwined with genetic drivers, most notably the loss of function of the von Hippel–Lindau (VHL) gene—a hallmark alteration in ccRCC [6]. VHL inactivation stabilizes hypoxia-inducible factors (HIFs), triggering a cascade of metabolic adaptations, including a shift toward aerobic glycolysis (the Warburg effect) even under oxygen-replete conditions [7]. Key glycolytic enzymes such as hexokinase 2 (HK2) and pyruvate kinase M2 (PKM2) are upregulated, diverting glucose away from oxidative phosphorylation toward rapid ATP production and shuttling glycolytic intermediates into biosynthetic pathways for nucleotide, lipid, and amino acid synthesis—critical for unchecked proliferation [8–10].

Beyond glycolysis, RCC exhibits a striking dependency on glutaminolysis. Tumor cells avidly uptake glutamine, which is catabolized via glutaminase (GLS1) to fuel the tricarboxylic acid (TCA) cycle through anaplerotic reactions, while also generating NADPH to maintain redox homeostasis [11]. This reliance on glutamine not only supports energy production but also buffers against oxidative stress, a common challenge in the tumor microenvironment [11, 12]. Additionally, de novo lipid synthesis is frequently hyperactive in RCC, driven by elevated expression of fatty acid synthase (FASN) and acetyl-CoA carboxylase (ACC), leading to lipid accumulation that contributes to membrane biogenesis and signaling cascades involved in tumor progression and drug resistance [10, 13]. The pentose phosphate pathway (PPP) also plays a pivotal role, with its activation supplying ribose-5-phosphate for nucleotide synthesis and NADPH for antioxidant defense, enhancing tumor cell survival under metabolic stress and chemotherapeutic pressure [14].

Transketolase (TKT), a pivotal metabolic enzyme in the non-oxidative phase of the PPP, serves as a central regulator in cellular metabolic networks [15]. The PPP, a critical branch of glucose metabolism, operates in concert with glycolysis to maintain cellular energy homeostasis and support biosynthetic processes [16]. By utilizing hexose phosphates and ketose phosphates as substrates, TKT orchestrates the redistribution of carbon skeletons, thereby ensuring metabolic flexibility and sustaining biosynthetic demands [16, 17]. Notably, ribose-5-phosphate (R5P), generated via TKT-mediated reactions, acts as a direct precursor for nucleotide biosynthesis, providing essential substrates for cellular proliferation, DNA replication, and repair mechanisms [15, 18]. Furthermore, TKT plays a crucial role in modulating intracellular levels of NADPH [19]. As a key reducing equivalent, NADPH drives anabolic reactions and safeguards redox balance, offering critical protection against oxidative stress [20, 21].

In recent years, TKT has become a key focus in tumor metabolism research [22, 23]. Evidence shows its pathological overexpression in various cancers, indicating its role in oncogenesis [16, 24–26]. High expression of TKT has been demonstrated to promote tumor progression and correlate with poor clinical outcomes [16, 27], whereas TKT ablation upregulates tumor suppressor pathways, suppresses oncometabolite accumulation, and effectively inhibits metastatic dissemination [17, 25, 27, 28], suggesting its potential as a therapeutic target [26, 29–31]. Nevertheless, research on TKT in RCC remains scare, necessitating further exploration of its role in RCC pathogenesis and its potential as a biomarker or therapeutic target.

Pyruvate kinase M2 (PKM2), a pivotal regulator of glycolysis, is commonly overexpressed and exhibits altered enzymatic activity in cancer cells, where it plays a central role in mediating aerobic glycolysis (the Warburg effect) [30]. As both TKT and PKM2 serve as crucial metabolic nodes in the interconnected PPP and glycolytic cascade respectively, their potential functional interplay warrants investigation. Emerging evidence suggests that metabolic reprogramming in malignant cells represents a highly integrated process characterized by extensive crosstalk between the PPP and glycolytic pathways [31, 32]. Mechanistically, TKT-derived PPP metabolites may regulate glycolytic flux, while PPP-generated R5P could modulate PKM2 expression and/or activity through two distinct mechanisms: (1) by serving as a substrate for nucleotide biosynthesis, and (2) by influencing downstream signaling cascades [33].

Treatment strategies of RCC vary by tumor stage and subtype. Localized disease is primarily treated with partial or radical nephrectomy, while advanced cases rely on systemic therapies like targeted treatments [34, 35], immunotherapy [3, 36–38] and cytotoxic chemotherapeutics [39]. However, targeted therapies face issues like intrinsic and acquired resistance [40, 41] in RCC and RCC shows low sensitivity to conventional cytotoxic agents, typifying primary chemoresistance, highlighting the need for new therapeutic targets and personalized treatment strategies [34, 35, 38, 42, 43].

In this study, we demonstrate that TKT is significantly upregulated in RCC and is strongly correlated with poor prognosis in RCC patients. Mechanistically, TKT reprograms the metabolic landscape of RCC cells by enhancing glycolysis, lactate production, and ATP generation, thereby facilitating tumor progression and metastasis. Furthermore, we identify a functional interplay between TKT and pyruvate kinase M2 (PKM2), which collaboratively promotes RCC progression and metastatic dissemination. These findings provide novel insights into the molecular mechanisms underlying dysregulated glucose metabolism in RCC and highlight the potential of TKT as a promising therapeutic target for RCC treatment.

## Results

### High expression of TKT correlates with advanced tumor characteristics and poor prognosis in RCC patients

**Fig. 1:**
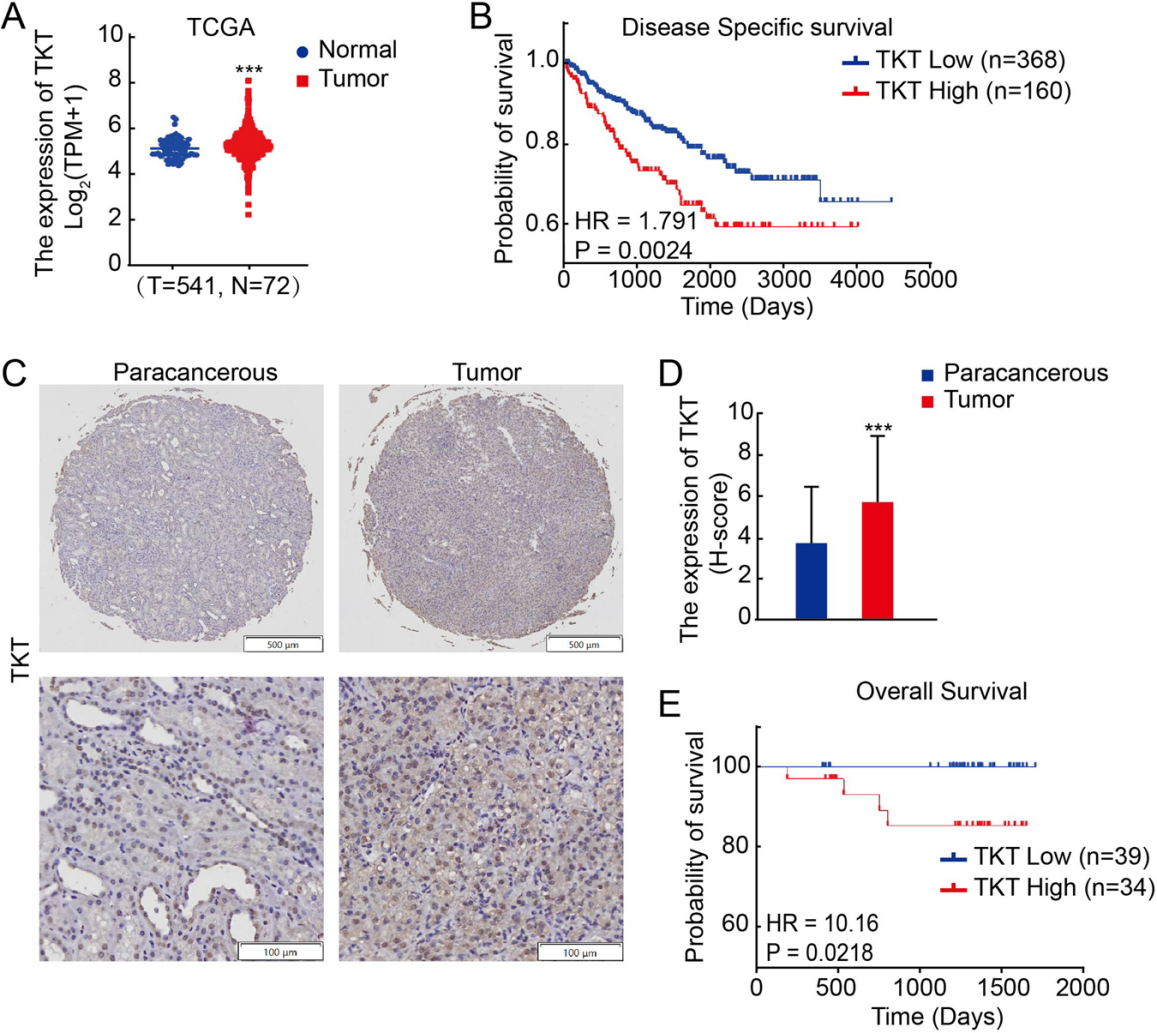
TKT is highly expressed in RCC tissues and correlates with poor clinical outcomes. **A** Analysis of TKT mRNA expression in renal carcinoma tissues (*n* = 541) compared with normal tissues (*n* = 72) based on the TCGA database. **B** Kaplan–Meier survival curves depicting disease-specific survival in RCC patients (*n* = 528, *P* = 0.0024) from the TCGA dataset. **C** Representative immunohistochemistry (IHC) images showing TKT protein expression in paracancerous and tumor tissues in RCC patients. **D** Quantification of TKT staining intensity in renal carcinoma tissues compared to adjacent normal tissues (*P* < 0.001). **E** Kaplan–Meier survival curves depicting overall survival (*n* = 73, *P* = 0.0218) stratified by TKT protein expression in RCC tissues. **P < 0.01, ***P < 0.001.

Through an analysis of data from the TCGA database, we observed that TKT was not only highly expressed in tumor tissues from kidney cancer patients (Fig. 1A), but also exhibited a negative correlation with patient survival. Specifically, patients with high TKT expression demonstrated worse disease specific survival (Fig. 1B). To further validate these findings, we examined TKT expression in tissue microarrays (TMAs) derived from 80 RCC patients. Immunohistochemical (IHC) analysis confirmed that TKT was significantly overexpressed in tumor tissues (Fig. 1C, D).

We subsequently assessed the correlation between TKT expression and various clinicopathological characteristics using Fisher’s exact test. The results, as presented in Table 1, indicated that TKT expression was not associated with patient gender or age. However, it demonstrated a significantly correlation with tumor size (*P* = 0.0265), TNM stage (*P* = 0.0015), and Tumor grade (*P* = 0.0003).

**Table 1:** TKT staining and clinicopathological characteristics of 74 renal cancer patients.

Variables	TKT staining			
	Low (%)	High (%)	Total	*P*^a^
All cases	40 (54.05)	34 (45.95)	74	
Age				
≤56	22 (64.71)	12 (35.29)	34	0.0659
>56	17 (42.50)	23 (57.50)	40	
Gender				
Male	27 (46.00)	23 (54.00)	50	0.8067
Female	12 (50.00)	12 (50.00)	24	
Tumor size				
≤7 cm	31 (62.00)	19 (38.00)	50	0.0265
>7 cm	8 (33.33)	16 (66.67)	24	
TNM stage				
T1	32 (66.67)	16 (33.33)	48	0.0015
T2–T4	7 (26.92)	19 (73.08)	26	
Tumor Grade				
Ⅰ	32 (69.57)	14 (30.43)	46	0.0003
Ⅱ-Ⅳ	7 (25.00)	21 (75.00)	28	

^a^*P* values are from χχ2 test.

To further investigate potential link between TKT expression and patient prognosis, we conducted a Kaplan–Meier survival analysis utilizing the follow-up data from RCC tumor samples. The survival analysis revealed that patients exhibiting high TKT expression had significantly poorer overall survival (OS) compared to those with low TKT expression (*P* = 0.0218) (Fig. 1E).

### TKT promotes RCC metastasis by enhancing cell migration and invasion in vitro and in vivo

**Fig. 2:**
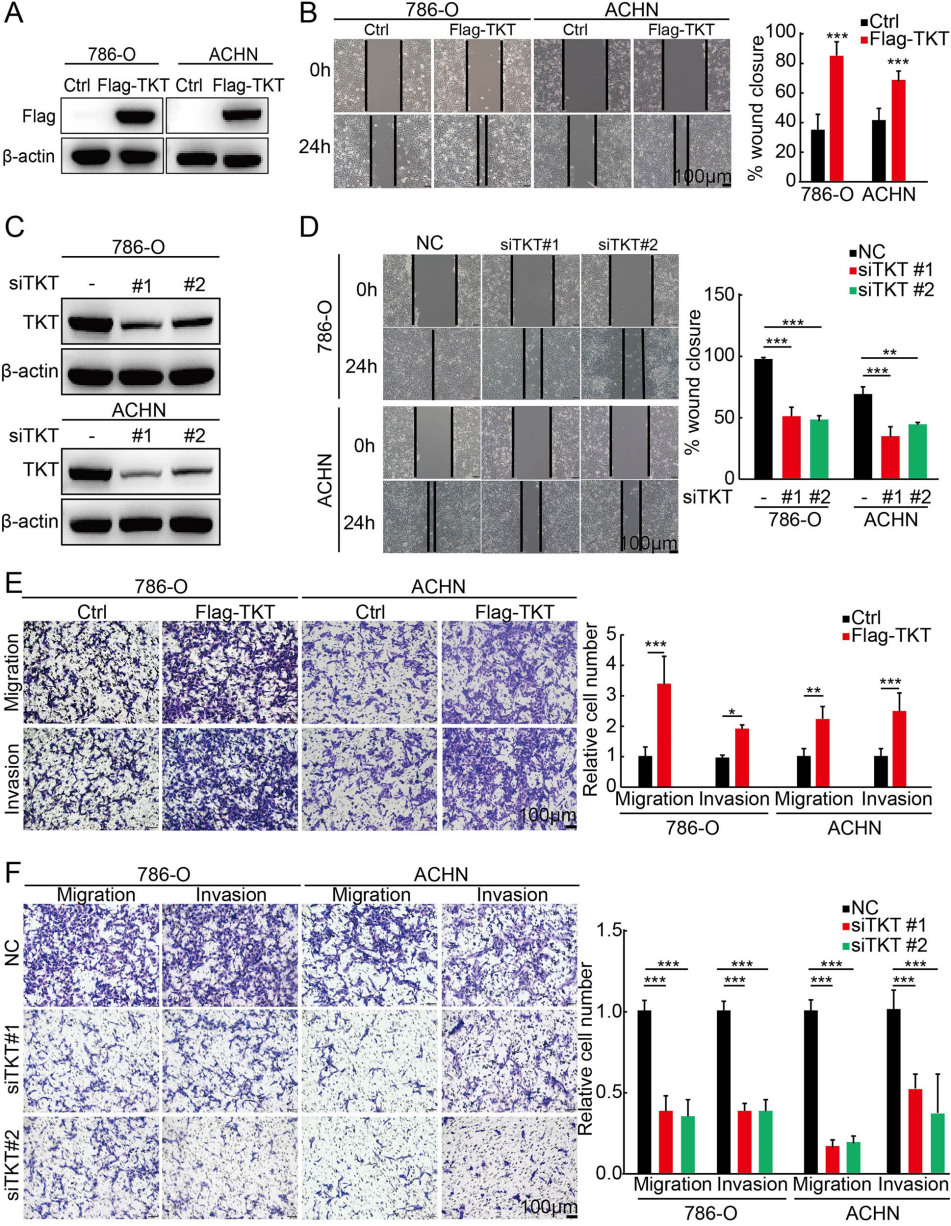
TKT promotes RCC cell migration and invasion in vitro. **A** Confirmation of TKT overexpression in 786-O and ACHN cells by western blot. **B** Wound healing assays indicating that TKT overexpression enhances RCC cell migration. **C** Confirmation of TKT knockdown in 786-O and ACHN cells by western blot. **D** Wound healing assays demonstrating that TKT knockdown significantly inhibits wound closure in 786-O and ACHN cells. **E** Transwell assays revealing that TKT overexpression significantly promotes migration and invasion in 786-O and ACHN cells, with corresponding statistical analyses. **F** Transwell assays showing that TKT knockdown reduces RCC cell motility, with corresponding statistical analyses. *P < 0.05, **P < 0.01, ***P < 0.001.

Given the positive correlation between high TKT expression and RCC patient’s outcome, we further investigated the impact of TKT on the migration and invasion of RCC by manipulating its expression in RCC cells. The wound-healing assay demonstrated that overexpression of TKT in 786-O and ACHN cells enhanced wound healing in RCC cells (Fig. 2A, B). Conversely, TKT knockdown restricted the wound healing ability (Fig. 2C, D). Additionally, cell migration and invasion assays confirmed this phenomenon (Fig. 2E, F).

**Fig. 3:**
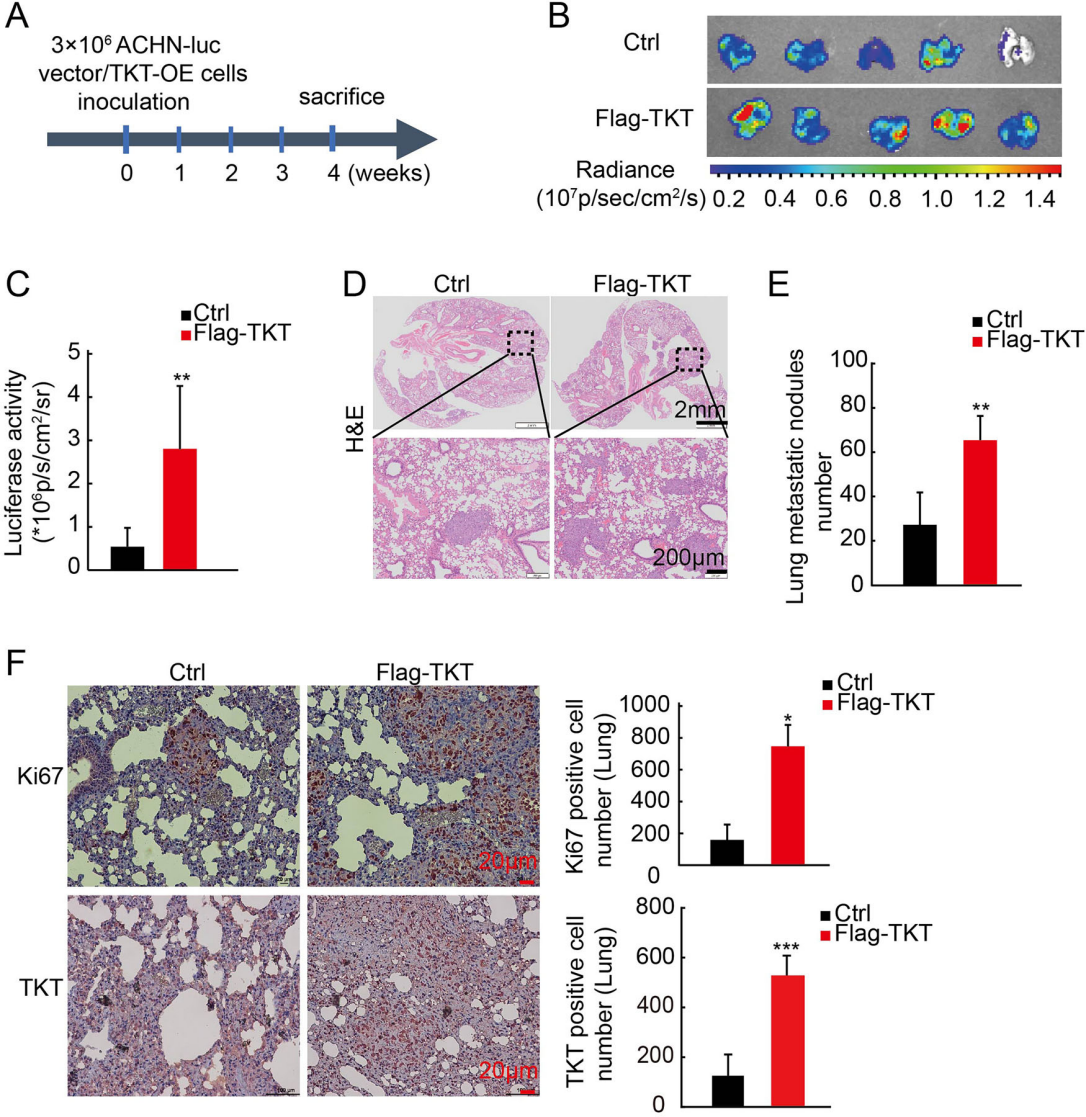
TKT promotes RCC metastasis in vivo. **A** Schematic representation of the experimental protocol for establishing a mouse tail vein lung metastasis model using vector/TKT-overexpressing ACHN-luc cells. **B**, **C** Lung bioluminescence imaging and statistical analysis of metastatic burden across different groups. **D** Representative Hematoxylin and Eosin (H&E)-stained lung sections of different groups are shown. **E** Quantification of lung metastatic nodules at week 4. **F** Ki-67 IHC analysis in the TKT-overexpressing lung metastasis model, with quantification of Ki-67-positive cells. *P < 0.05, **P < 0.01, ***P < 0.001.

These findings suggest that TKT plays a critical role in the migration and invasion of RCC cells, potentially contributing to the metastatic progression of renal cancer. To validate this hypothesis, we established a tumor metastasis model by injecting 3 million luciferase-labeled TKT-overexpressing or control ACHN cells into the systemic circulation of mice via the tail vein. After 4 weeks, the mice were sacrificed, and their lung tissues were collected for analysis. The experimental workflow for the mouse lung metastasis model is illustrated in Fig. 3A. Bioluminescence imaging of lung tissues revealed that the fluorescence intensity in the TKT overexpression group (Flag-TKT) was significantly higher than that in the control group (Ctrl) (Fig. 3B, C). Furthermore, the TKT overexpression group exhibited a greater number of lung metastases compared to the control group (Fig. 3D, E). Additionally, increased Ki-67 expression was observed in lung tissues from the TKT overexpression group in the mouse tumor metastasis model (Fig. 3F).

Collectively, these data confirm that TKT enhances the metastatic potential of tumor cells, thereby promoting the malignant progression of renal cancer by improving the migration and invasion capabilities of RCC cells.

### TKT accelerates RCC progression by promoting tumor cell proliferation and growth in vitro and in vivo

**Fig. 4:**
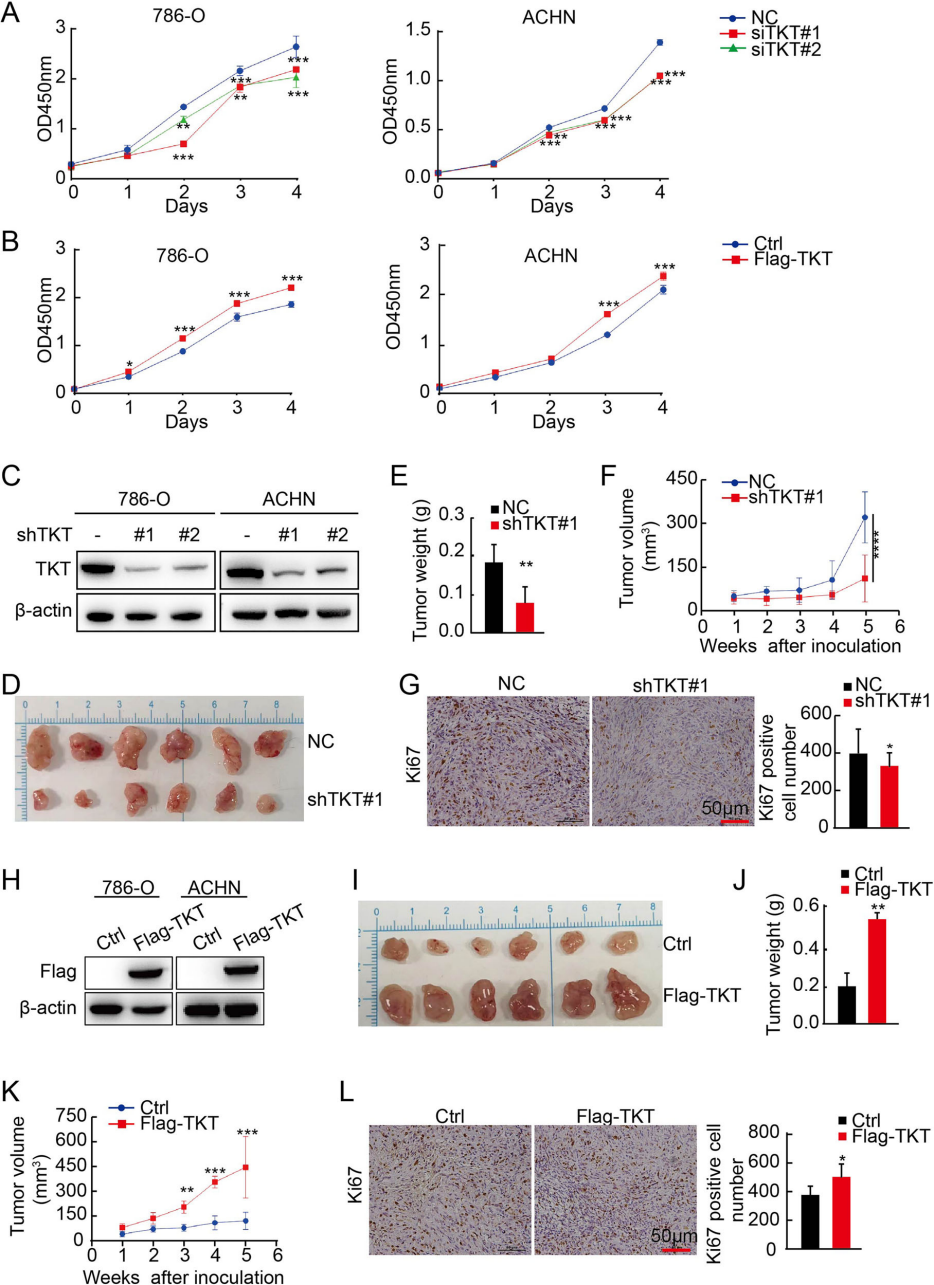
TKT accelerates RCC cell proliferation in vitro and in vivo. **A**, **B** Cell Counting Kit-8 (CCK-8) assays assessing the impact of TKT knockdown/ overexpression on RCC cell proliferation in 786-O and ACHN cells. **C** Western blot validation of TKT knockdown in stable 786-O and ACHN cell lines. **D**–**F** Effect of TKT knockdown in ACHN cells on the xenograft model was assessed by evaluating tumor weight and tumor volume. **G** IHC analysis of Ki-67 expression and quantification of Ki-67-positive cells in TKT-overexpressing xenograft tumors. **H** Western blot validation of TKT overexpression in stable 786-O and ACHN cell lines. **I**–**K** Effect of TKT overexpression in ACHN cells on the xenograft model was assessed by evaluating tumor weight and tumor volume. **L** Ki-67 IHC analysis in the TKT-overexpressing lung metastasis model, with quantification of Ki-67-positive cells. *P < 0.05, **P < 0.01, ***P < 0.001.

The malignant progression of tumor cells necessitates substantial bioenergetic support [44, 45]. As the rate-limiting enzyme in the non-oxidative branch of the PPP, TKT plays a pivotal role in facilitating the exchange of metabolites between glycolysis and the PPP [19]. This enzymatic activity is crucial for providing ATP and biosynthetic precursors essential for tumor cell survival and proliferation [46]. To investigate whether elevated TKT expression could promote RCC proliferation, we conducted cell proliferation assays using the Cell Counting Kit-8 (CCK-8) assay. The results showed that TKT knockdown in 786-O and ACHN cells significantly inhibited RCC cell proliferation (Fig. 4A), while TKT overexpression markedly enhanced renal carcinoma cell proliferation (Fig. 4B).

To further elucidate the role of TKT in tumor growth in vivo, we established xenograft models. Stable TKT knockdown were generated in 786-O and ACHN cells (Fig. 4C), which the ACHN shTKT cells were subsequently subcutaneously injected into nude mice. The results revealed that tumor size and weight were significantly reduced in the shTKT group compared to the control group (Fig. 4D, E), with a notably slower tumor growth curve (Fig. 4F). Additionally, the number of Ki-67-positive cells in tumors from the shTKT group was decreased (Fig. 4G). Conversely, in the TKT overexpression group, tumor size and weight were significantly increased compared to the vector control group (Fig. 4H–J), and the tumor growth rate was markedly accelerated (Fig. 4K). Furthermore, the number of Ki-67-positive cells in tumors from the TKT overexpression group was elevated (Fig. 4L), consistent with the in vitro results.

These findings underscore that TKT promotes RCC progression by enhancing tumor cell proliferation in vitro and accelerating tumor growth, Ki-67 expression, and metastatic potential in vivo, as demonstrated by xenograft models.

### TKT promotes RCC growth and progression by enhancing glucose uptake, lactate production, and ATP generation

Tumorigenesis and cancer progression are critically dependent on the metabolic reprogramming of tumor cells [44]. A substantial proportion of tumor metabolites, such as lactate, which is predominantly generated via glycolysis, are redirected into anabolic pathways to sustain tumor cell proliferation and facilitate tumor growth and metastasis [47–49]. As a key metabolic enzyme in the non-oxidative phase of the PPP, TKT may influence RCC initiation and progression by modulating glycolysis. To investigate this possibility, we examined the effects of aberrant TKT expression on glucose metabolism and lactate production in RCC cells with levels of TKT expression.

**Fig. 5:**
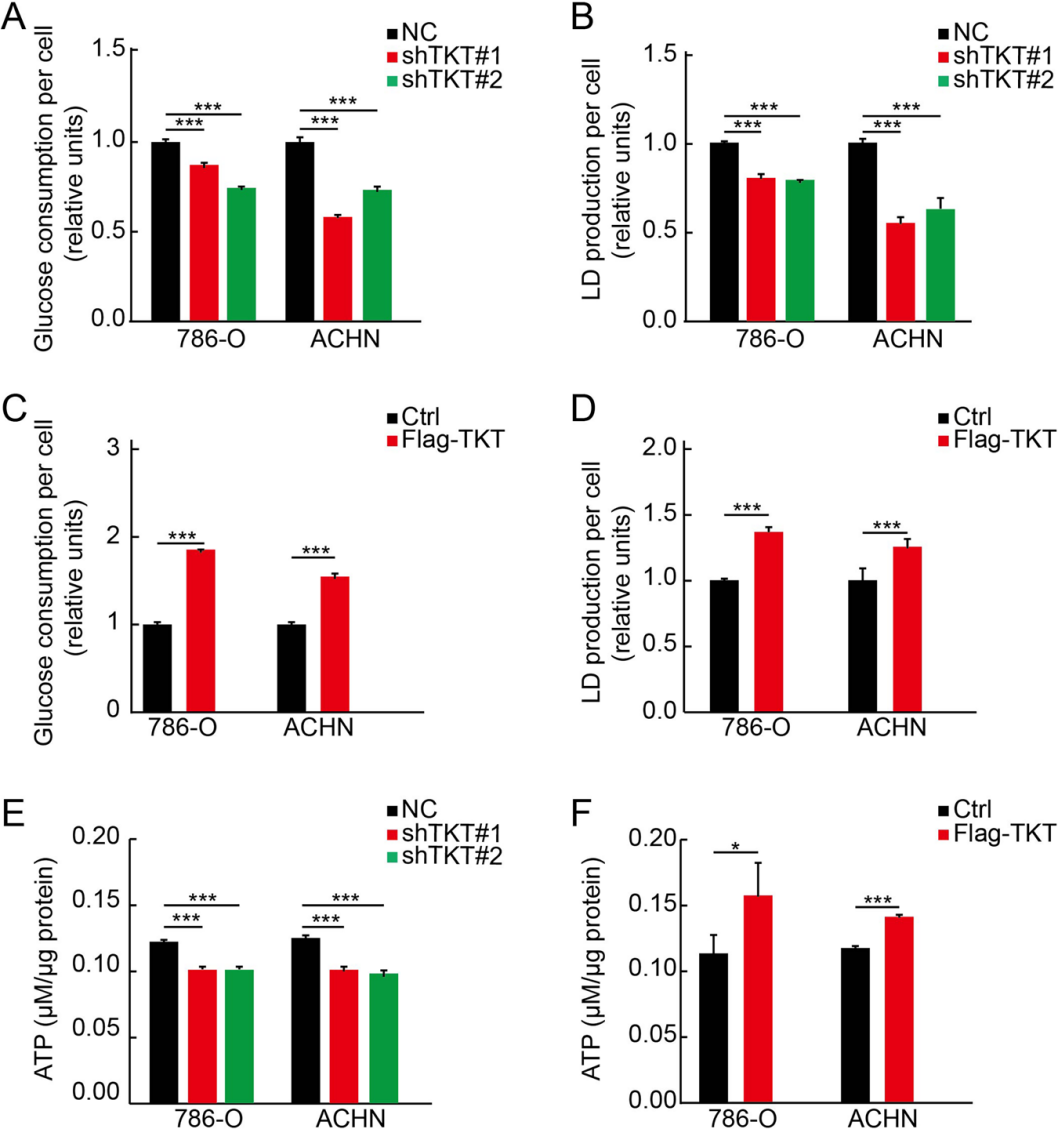
TKT promoted aerobic glycolysis of RCC cells. **A** Glucose uptake assays showed that TKT knockdown significantly inhibited aerobic glycolysis. **B** TKT knockdown leaded to decreased lactate production. **C** Glucose uptake assays showed that TKT overexpression significantly promoted aerobic glycolysis. **D** TKT overexpression leaded to increased lactate production. **E** TKT knockdown leaded to decreased ATP production. **F** TKT overexpression leaded to increased ATP production. *P < 0.05, **P < 0.01, ***P < 0.001.

Our findings revealed that TKT knockdown significantly reduced glucose uptake (Fig. 5A) and lactate production (Fig. 5B) in RCC cells. In contrast, TKT overexpression markedly enhanced glucose uptake (Fig. 5C) and significantly increased lactate production (Fig. 5D). These results indicate that TKT overexpression facilitates glycolysis in RCC cells by promoting glucose uptake.

Although tumor cells preferentially rely on glycolysis for energy production, even under aerobic conditions, this metabolic pathway is relatively inefficient, yielding only two ATP molecules per glucose molecule [50]. Furthermore, enhanced glycolysis does not necessarily correlate with increased ATP production across all tumor types [51, 52]. To investigate whether TKT-driven glycolysis in RCC also promotes ATP generation, we measured ATP levels in RCC cells with altered TKT expression. The results showed that ATP production was significantly reduced following TKT inhibition (Fig. 5E), while TKT overexpression resulted in a marked increase in ATP levels (Fig. 5F).

Collectively, these findings underscore the role of TKT as a key metabolic regulator in RCC. By enhancing glucose uptake, lactate production, and ATP generation, TKT reprograms glucose metabolism to facilitate tumor growth and progression. These insights further establish TKT as a potential therapeutic target for RCC treatment.

### Regulation of TKT to aerobic glycolysis is dependent on PKM2

**Fig. 6:**
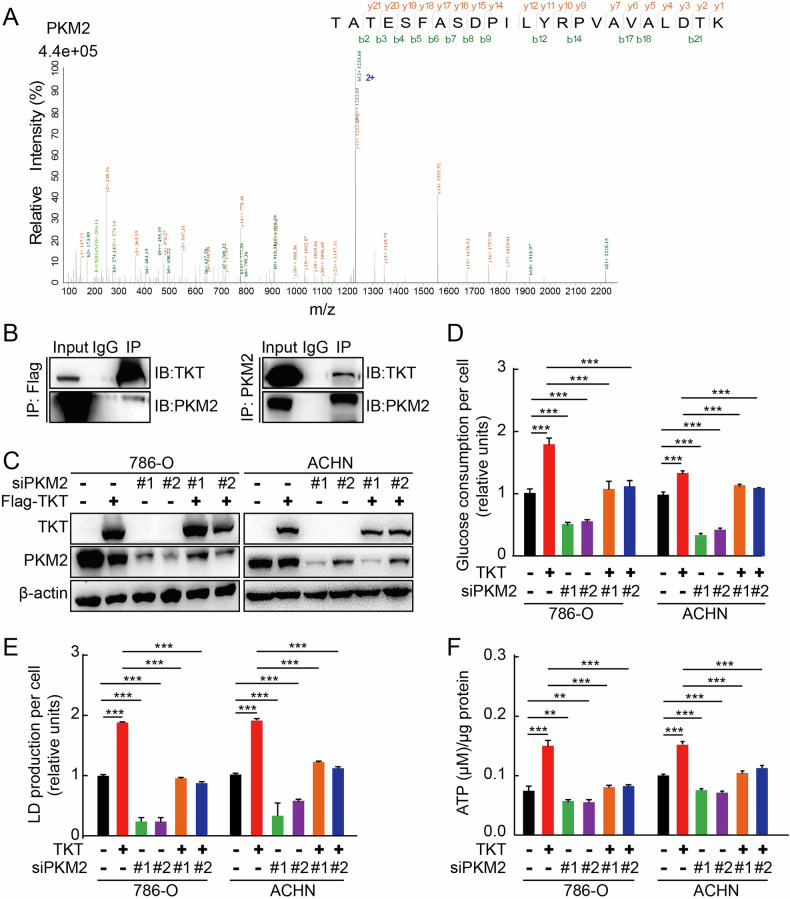
Regulation of TKT to aerobic glycolysis is dependent on PKM2. **A** Representative images from Immunoprecipitation-Mass Spectrometry (IP-MS) analysis, demonstrating TKT-PKM2 interaction. **B** TKT coimmunoprecipitates with PKM2 in ACHN. **C** Western blot confirming PKM2 knockdown in control and TKT-overexpressing 786-O/ACHN cells. **D**–**F** Regulation of TKT to aerobic glycolysis is dependent on PKM2. *P < 0.05, **P < 0.01, ***P < 0.001.

To investigate the possible mechanism of TKT in aerobic glycolysis, we performed mass spectrometry analysis of TKT-binding proteins in RCC cells and focused on PKM2 (Fig. 6A). PKM2 is a well-established glycolytic enzyme that catalyzes the final step of glycolysis, generating ATP. In tumors, PKM2 plays a pivotal role in aerobic glycolysis, favoring glycolysis even in oxygen-rich conditions to support tumor growth and progression [53, 54].

This interaction was further validated by co-immunoprecipitation assays (Fig. 6B), confirming a direct association between TKT and PKM2.

Next, we examined whether TKT and PKM2 cooperatively regulate RCC aerobic glycolysis. We designed a small interfering RNA (siRNA) targeting PKM2 and transfected it into renal cancer cells overexpressing TKT (Fig. 6C). PKM2 depletion markedly suppressed the TKT-induced increase in glucose uptake (Fig. 6D) and lactate production (Fig. 6E). Moreover, ATP levels were significantly reduced upon PKM2 knockdown in TKT-overexpressing RCC cells (Fig. 6F), indicating that PKM2 is essential for the metabolic effects driven by TKT. Conversely, upon overexpressing PKM2 in renal cancer cells with stable TKT knockdown (Supplementary Fig. S1A), we observed that such overexpression reversed the TKT knockdown-induced metabolic alterations, leading to elevated glucose uptake (Supplementary Fig. S1B), lactate production (Supplementary Fig. S1C), and ATP levels (Supplementary Fig. S1D). These results indicated that the regulation of TKT on glycolysis was dependent on PKM2.

### Regulation of TKT to RCC cells metastasis and proliferation is dependent on PKM2

**Fig. 7:**
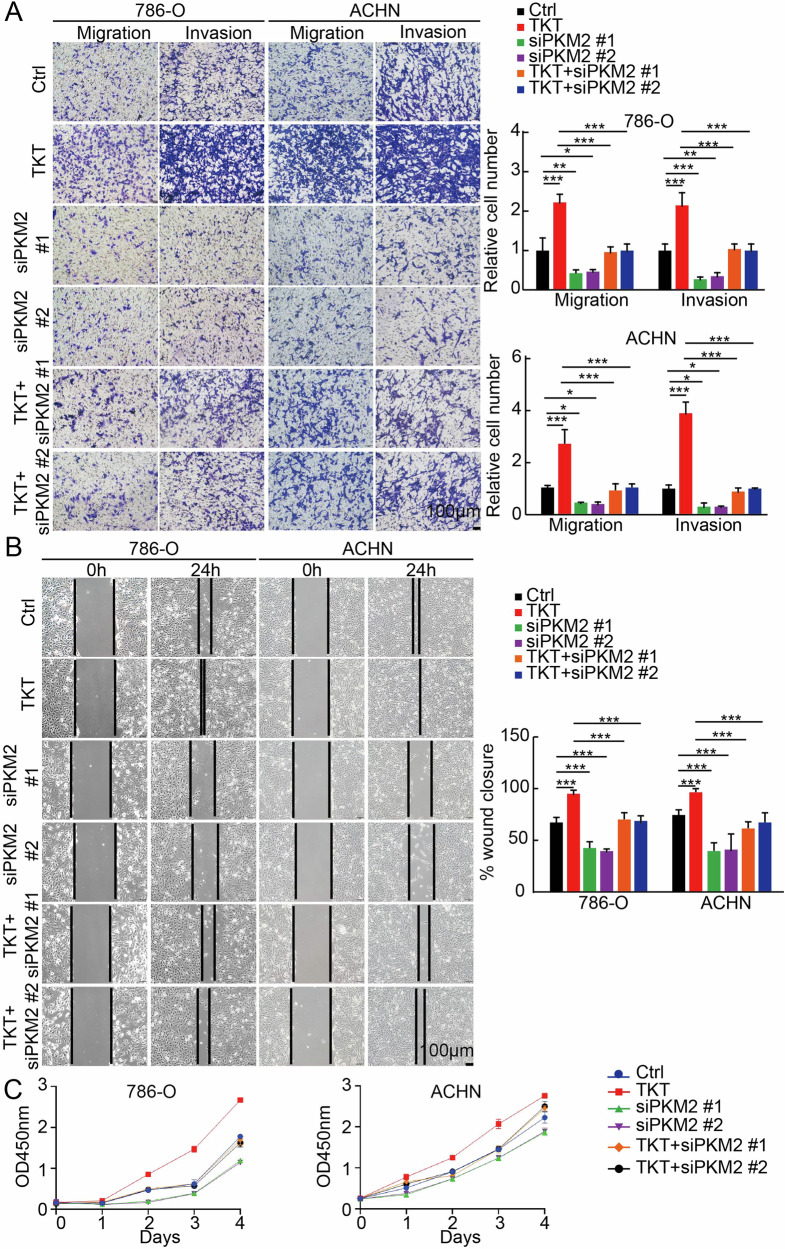
Regulation of TKT to RCC cells metastasis and proliferation is dependent on PKM2. **A** Transwell assays assessing the impact of PKM2 knockdown on TKT-driven RCC cell migration, invasion. **B** Wound healing assays demonstrating that PKM2 knockdown significantly reverses TKT-driven wound closure in RCC cells. **C** CCK-8 assays assessing the impact of PKM2 knockdown on TKT-driven RCC cell proliferation. Relative statistical results of CCK-8 assays are as following. In 786-O cells, Day 2: Ctrl vs TKT: ***; Ctrl vs siPKM2 #1: ***; Ctrl vs siPKM2 #2: ***; TKT vs TKT+ siPKM2 #1: ***; TKT vs TKT+ siPKM2 #2: ***. Day 3: Ctrl vs TKT: ***; Ctrl vs siPKM2 #1: ***; Ctrl vs siPKM2 #2: ***; TKT vs TKT+ siPKM2 #1: ***; TKT vs TKT+ siPKM2 #2: ***. Day 4: Ctrl vs TKT: ***; Ctrl vs siPKM2 #1: ***; Ctrl vs siPKM2 #2: ***; TKT vs TKT+ siPKM2 #1: ***; TKT vs TKT+ siPKM2 #2: ***. In ACHN cells, Day 2: Ctrl vs TKT: ***; Ctrl vs siPKM2 #1: ***; Ctrl vs siPKM2 #2: ***; TKT vs TKT+ siPKM2 #1: ***; TKT vs TKT+ siPKM2 #2: ***. Day 3: Ctrl vs TKT: ***; Ctrl vs siPKM2 #1: ***; Ctrl vs siPKM2 #2: ***; TKT vs TKT+ siPKM2 #1: ***; TKT vs TKT+ siPKM2 #2: ***. Day 4: Ctrl vs TKT: ***; Ctrl vs siPKM2 #1: ***; Ctrl vs siPKM2 #2: ***; TKT vs TKT+ siPKM2 #1: ***; TKT vs TKT+ siPKM2 #2: ***. *P < 0.05, **P < 0.01, ***P < 0.001.

Based on the results obtained, we subsequently assessed whether the regulation of TKT on the metastasis and proliferation of RCC cells is dependent on PKM2. Migration and invasion assays demonstrated that PKM2 knockdown significantly reduced the enhanced migratory and invasive capabilities induced by TKT overexpression (Fig. 7A). Furthermore, the wound-healing assay corroborated these findings (Fig. 7B). Similarly, cell proliferation assays indicated that silencing PKM2 mitigated the TKT-mediated increase in RCC cell proliferation (Fig. 7C). Consistent with the above findings, ectopic overexpression of PKM2 in renal carcinoma cells with stable TKT knockdown effectively rescued the inhibitory effects of TKT deficiency on both metastatic (Supplementary Fig. S2A) and proliferative capacity (Supplementary Fig. S2B, C) potential.

Moreover, both genetic silencing and overexpression of PKM2 significantly influenced RCC proliferation and metastatic potential by orchestrating metabolic reprogramming. These findings are consistent with previous studies that demonstrate PKM2’s role in regulating lactate production and secretion, which contributes to the acidification of the tumor microenvironment, subsequently promoting tumor invasion and immune evasion [55, 56]. Given these mechanisms, we hypothesize that the dual knockdown of TKT and PKM2 could synergistically enhance the suppression of tumor progression.

### TKT modulates cisplatin sensitivity in RCC cells via regulation of PKM2

RCC cells, particularly ccRCC, generally display low sensitivity to conventional cytotoxic chemotherapeutic agents such as cisplatin [57]. This reduced sensitivity potentially linked to the unique metabolic traits of RCC as well as drug resistance-associated mechanisms [58, 59]. Given the established role of the TKT-PKM2 axis in mediating metabolic reprogramming in RCC cells, we further investigated whether blocking this axis could augment the sensitivity of RCC cells to chemotherapy. To this end, we assessed RCC cell viability under cisplatin treatment.

**Fig. 8:**
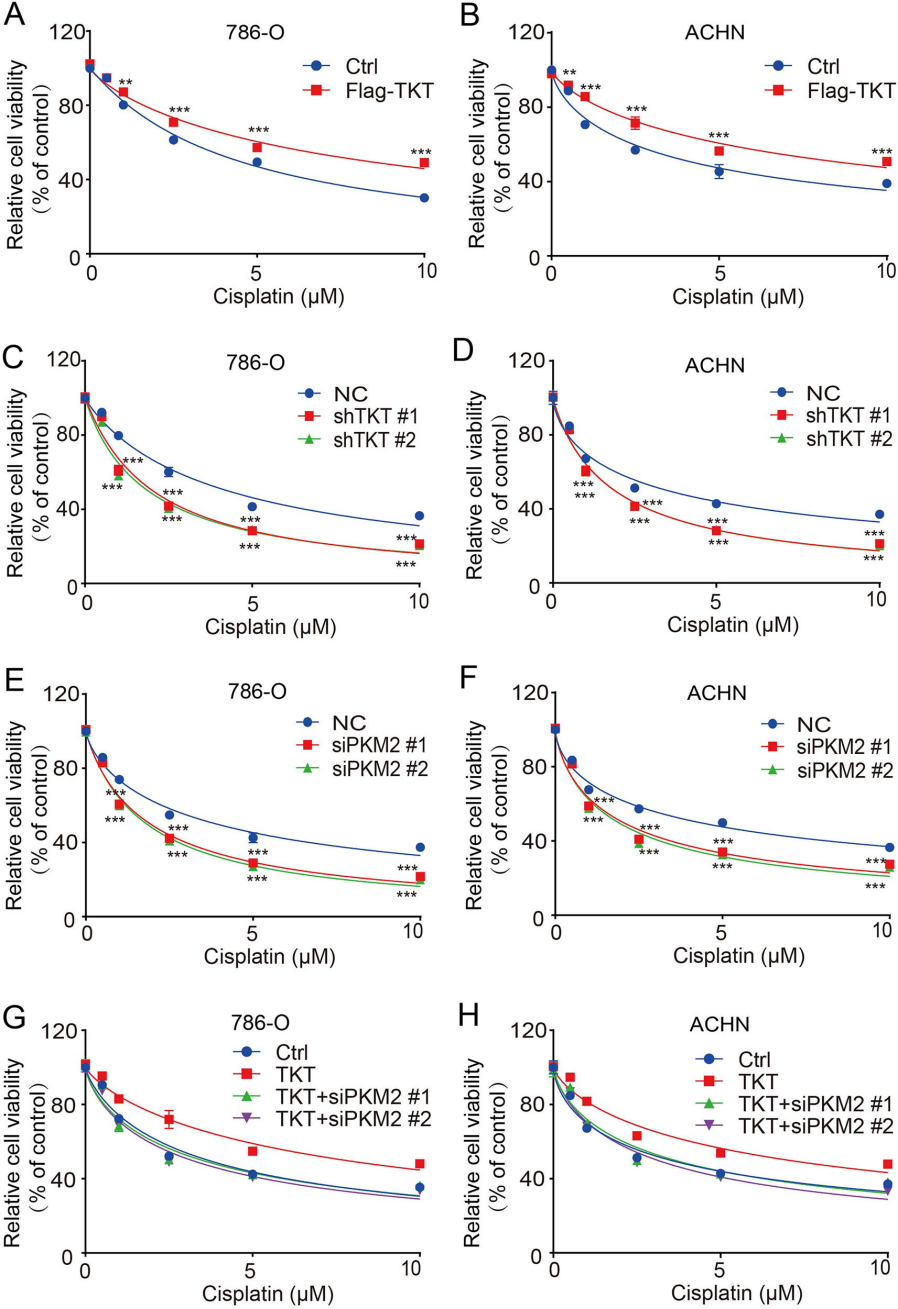
The sensitivity of RCC cells to cisplatin can be enhanced by blocking the TKT-PKM2 axis. **A**, **B** CCK-8 assays showing the effect of TKT overexpression on the viability of 786-O and ACHN cells treated with cisplatin at different concentrations. **C**, **D** CCK-8 assays demonstrating the effect of TKT knockdown on the viability of 786-O and ACHN cells following treatment with cisplatin at different concentrations. **E**, **F** CCK-8 assays illustrating the effect of PKM2 knockdown on the viability of 786-O and ACHN cells upon treatment with cisplatin at different concentrations. **G**, **H** CCK-8 assays showing the effect of PKM2 knockdown on the viability of TKT overexpressed 786-O and ACHN cells upon treatment with cisplatin at different concentrations. In 786-O cells, 0.5 µM: Ctrl vs TKT: *; TKT vs TKT+ siPKM2 #1: *; TKT vs TKT+ siPKM2 #2: ***. 1 µM: Ctrl vs TKT: ***; TKT vs TKT+ siPKM2 #1: ***; TKT vs TKT+ siPKM2 #2: ***. 2.5 µM: Ctrl vs TKT: ***; TKT vs TKT+ siPKM2 #1: ***; TKT vs TKT+ siPKM2 #2: ***. 5 µM: Ctrl vs TKT: ***; TKT vs TKT+ siPKM2 #1: ***; TKT vs TKT+ siPKM2 #2: ***. 10 µM: Ctrl vs TKT: ***; TKT vs TKT+ siPKM2 #1: ***; TKT vs TKT+ siPKM2 #2: ***. In ACHN cells, 0.5 µM: Ctrl vs TKT: ***; TKT vs TKT+ siPKM2 #1: ***; TKT vs TKT+ siPKM2 #2: ***. 1 µM: Ctrl vs TKT: ***; TKT vs TKT+ siPKM2 #1: ***; TKT vs TKT+ siPKM2 #2: ***. 2.5 µM: Ctrl vs TKT: ***; TKT vs TKT+ siPKM2 #1: ***; TKT vs TKT+ siPKM2 #2: ***. 5 µM: Ctrl vs TKT: ***; TKT vs TKT+ siPKM2 #1: ***; TKT vs TKT+ siPKM2 #2: ***. 10 µM: Ctrl vs TKT: ***; TKT vs TKT+ siPKM2 #1: ***; TKT vs TKT+ siPKM2 #2: ***. *P < 0.05, **P < 0.01, ***P < 0.001.

As illustrated in Fig. 8A, B, TKT overexpression in RCC cells resulted in a significant increase in relative cell viability following cisplatin exposure, indicating that TKT overexpression confers cisplatin resistance in RCC cells. In contrast, TKT knockdown in RCC cells led to a marked reduction in relative viability (Fig. 8C, D), suggesting that TKT depletion enhances RCC cell sensitivity to cisplatin.

We further evaluated the impact of PKM2 knockdown on cisplatin sensitivity. As shown in Fig. 8E, F, PKM2 knockdown induced a significant decrease in cell viability, demonstrating that PKM2 depletion also enhances cisplatin sensitivity in RCC cells. Consistent with the above findings demonstrating that TKT exerts its regulatory effects in a PKM2-dependent manner, we further performed combinatorial experiments involving TKT overexpression and PKM2 knockdown. Results revealed that the cell viability in the TKT-overexpressing group with concurrent PKM2 knockdown was significantly reduced compared to that in the TKT-overexpression alone group, and notably, it was restored to a level comparable to that of the control group (Fig. 8G, H). These observations indicate that PKM2 knockdown can abrogate TKT overexpression-induced cisplatin resistance, thereby supporting the notion that TKT participates in the modulation of cisplatin sensitivity in renal cancer cells through the regulation of PKM2. Collectively, these findings indicate that TKT overexpression promotes cisplatin resistance, whereas knockdown of either TKT or PKM2 enhances RCC cell sensitivity to cisplatin, supporting the involvement of the TKT-PKM2 axis in regulating the cisplatin response in RCC.

In summary, our findings reveal that TKT collaborates with PKM2 to drive renal cancer metastasis by enhancing cell migration and invasion. Furthermore, by reprogramming glucose metabolism, TKT and PKM2 collectively sustain energy production, there by supporting the malignant proliferation and progression of RCC. These results provide new insights into the metabolic mechanisms underlying RCC progression and highlight the potential therapeutic value of targeting the TKT-PKM2 axis.

## Discussion

RCC is characterized by extensive metabolic reprogramming, a hallmark of cancer that facilitates tumor adaptation to its microenvironment and sustains rapid proliferation.

In this study, we provide compelling evidence that TKT expression is significantly elevated in RCC tissues and correlates with adverse clinical outcomes, including larger tumor size, advanced TNM stage, and reduced overall survival. These clinical correlations align with functional studies showing that TKT enhances RCC cell migration, invasion, and proliferation in vitro, as well as metastatic dissemination and tumor growth in vivo. These results extend prior observations of TKT’s oncogenic roles in other malignancies such as hepatocellular carcinoma [19] and breast cancer [60] to RCC. underscoring TKT as a key metabolic driver in this disease in RCC.

Notably, TKT’s pro-tumorigenic effects are linked to its ability to reprogram glucose metabolism. We found that TKT enhances glucose uptake, lactate production, and ATP generation—key hallmarks of aerobic glycolysis, commonly known as the “Warburg effect,” even in the presence of oxygen. TKT is essential for nucleotide biosynthesis and redox homeostasis, processes frequently dysregulated in cancer cells [19]. As a central enzyme in the non-oxidative phase of the PPP, TKT facilitates carbon redistribution between glycolysis and the PPP, ensuring a steady supply of biosynthetic precursors and reducing equivalents to support unchecked proliferation and redox homeostasis [61, 62]. This metabolic shift contributes to TKT-mediated tumor progression, particularly under the nutrient- and oxygen-deprived conditions of the RCC microenvironment.

A key mechanistic insight from our study is the identification of a functional interplay between TKT and PKM2, a master regulator of glycolysis, which further amplifies this metabolic shift. Co-immunoprecipitation and mass spectrometry analyses confirmed a direct interaction between these enzymes, and functional assays revealed that TKT’s effects on glycolysis, proliferation, and metastasis are strictly dependent on PKM2. Specifically, PKM2 knockdown abrogated TKT-induced increases in glucose uptake, lactate production, and ATP levels, while restoring PKM2 in TKT-deficient cells reversed these metabolic defects. Similarly, PKM2 depletion blocked TKT-mediated enhancements in cell migration, invasion, and proliferation, underscoring the interdependence of these two metabolic nodes.

This TKT-PKM2 axis likely operates through coordinated regulation of metabolic flux. PKM2 catalyzes the final step of glycolysis, generating ATP and pyruvate, while TKT shuttles intermediates between glycolysis and the PPP to support biosynthesis. Their interaction may facilitate substrate channeling or allosteric regulation, optimizing the balance between energy production (via glycolysis) and biosynthetic capacity (via PPP). Moreover, the cooperative regulation of RCC metabolism by TKT and PKM2 highlights a potential metabolic vulnerability in tumor cells. Disrupting this axis could compromise the metabolic adaptability of RCC cells, thereby impairing tumor growth and metastasis.

RCC’s intrinsic resistance to cytotoxic agents like cisplatin remains a major clinical hurdle [35, 63]. Our results show that TKT overexpression reduces cisplatin sensitivity, while knockdown of TKT or PKM2 enhances it. This suggests that the TKT-PKM2 axis contributes to chemoresistance, possibly by sustaining energy production for DNA damage repair or reducing oxidative stress. By disrupting this axis, we may impair the metabolic adaptations that enable RCC cells to survive cisplatin-induced stress-providing a rationale for combining TKT/PKM2 inhibitors with cisplatin to improve therapeutic efficacy.

These results highlight the therapeutic potential of targeting the TKT-PKM2 axis. Inhibiting TKT or PKM2 could disrupt the metabolic adaptations that sustain RCC progression and chemoresistance, and a dual-targeting approach against TKT and PKM2 may offer a more effective therapeutic strategy for RCC. Moreover, further investigation into the metabolic interplay between TKT and other key enzymes in RCC could reveal additional therapeutic targets.

In summary, our study identifies TKT as a prognostic marker and oncogenic driver in RCC, acting through a novel TKT-PKM2 axis to promote metabolic reprogramming, malignancy, and chemoresistance. These findings highlight the therapeutic potential of targeting TKT-PKM2, offering a promising strategy to improve outcomes for RCC patients, particularly those with advanced or treatment-resistant disease.

## Methods

### Tissue microarrays data

The RCC tissue microarray (TMA) slides were purchased from Hunan AiFang biological company. The slide contains 80 RCC tumor tissues and correspondingly 80 paracancer tissues. All these patients were treated with surgery only or with postoperative adjuvant therapy. The patients’ clinicopathologic information including age at diagnosis, gender, tumor size, tumor grade and lymph node metastasis, was obtained from the archive of the pathology department and confirmed by the medical record of the hospital. The tumor grade was assessed basing on TNM stage. Clinical follow-up results are available for 80 patients.

### Cell culture

RCC cell lines were obtained from the cell bank of the Chinese Academy of Sciences. ACHN and 786-O were cultured in DMEM and RPMI-1640 medium, respectively, supplemented with 10% fetal bovine serum, 100 U/ml penicillin, and 100 μg/ml streptomycin. All cells were cultured in a 37 °C humidified incubator with 5% CO_2_.

TKT and PKM2 Small interfering RNAs (siRNAs, 50 nM) were transfected into the RCC cell using jetPRIME reagent. Non-specific siRNA was used as negative controls. All siRNAs were purchased from GenePharma Technology (Shanghai, China). The siRNA sequences are as follows:

siTKT #1: sense, 5’- UUGGUGAGGACGAUUAUGGTT -3’; antisense, 5’-UUGUCCAGCUUAUAGAUGCTT-3’;

siTKT #2: sense, 5’-GCCGCCAAUACAAAGGGUATT-3’; antisense, 5’-UACCCUUUGUAUUGGCGGCTT-3’;

siPKM2 #1: sense, 5’-GAUUAAGUCUGGAAUGAAUTT-3’; antisense, 5’-AUUCAUUCCAGACUUAAUCTT-3’.

siPKM2 #2: sense, 5’-CCAUAAUCGUCCUCACCAATT-3’; antisense, 5’-UUGGUGAGGACGAUUAUGGTT-3’.

For plasmid transfections, cells were transfected using Lipofectamine 8000 (C0533, Beyotime Biotechnology, Shanghai, China), according to the manufacturer’s instructions.

### Stable cell line generation

We constructed the pCDH-CMV-MCS-EF1-GreenPuro-CD513B-TKT lentivirus plasmid, and purchased TKT short hairpin RNA (shRNA) (GenePharma, Shanghai, China). Stable cells were generated using lentivirus through a previously described method [64]. The shRNAs against human TKT and GRP78 were purchased from GenePharma Technology (Shanghai, China). shRNAs were listed in below:

shTKT-1:

GCCGCCAAUACAAAGGGUATTUACCCUUUGUAUUGGCGGCTT

shTKT-2:

CCGGCAAAUACUUCGACAATTUUGUCGAAGUAUUUGCCGGTT

### Western blot analysis, antibodies and reagents

Western blot analysis was performed as previously described [65]. The primary antibodies used in this study were as follows: transketolase (TKT; 1:1000 dilution, #11039-1-AP, Proteintech Group, Wuhan, China), pyruvate kinase M2 (PKM2; 1:1000 dilution, #15822-1-AP), β-actin (1:2000 dilution, #4970, Cell Signaling Technology, Danvers, MA, USA), and Flag tag (1:2000 dilution, sc-807, Santa Cruz Biotechnology, Dallas, TX, USA). The HRP-conjugated secondary antibodies were as follows: anti-rabbit IgG (#10828, Cell Signaling Technology, Danvers, MA, USA); anti-mouse IgG (#18030, Cell Signaling Technology, Danvers, MA, USA). All primary antibodies were diluted in a commercial antibody dilution buffer (WB100D, NCM Biotech, Suzhou, China) according to the manufacturer’s instructions and all secondary antibodies were diluted in 5%(w/v) non-fat milk in TBS.

### Immunoprecipitation

Cells were lysed using Cell Lysis Buffer for Western and IP (P0013, Beyotime Biotechnology, Shanghai, China) and incubated with primary antibodies at 4 °C overnight. Subsequently, protein A/G-agarose beads (HY-K0202, MedChemExpress, Monmouth Junction, NJ, USA) were added and incubated for an additional 2 h at 4 °C. The beads were washed three times with cell lysis buffer and resuspended in 1× SDS protein loading buffer, followed by boiling at 95 °C for 5 min. The immunoprecipitated proteins were then subjected to Western blot analysis.

### Cell proliferation

Cell proliferation was assessed using a Cell Counting Kit-8 (#40203ES80, Yeasen Biotechnology, Shanghai, China). Briefly, transfected 786-O and ACHN cells were seeded into 96-well plates at a density of 4 × 10³ cells/well and allowed to adhere overnight. At the indicated time points, 10 µL of CCK-8 solution was added to each well containing 100 µL of complete culture medium, followed by incubation at 37 °C for 1 h in a humidified 5% CO₂ atmosphere. Absorbance was measured at 450 nm using a Cytation3 microplate spectrophotometer (BioTek Instruments, Inc., Winooski, VT, USA). Each experiment was performed in triplicate, and background absorbance was corrected by subtracting the values from blank wells containing medium alone.

### Cell viability assays

Cell viability was assessed using the Cell Counting Kit-8 (#40203ES80, Yeasen Biotechnology, Shanghai, China), according to the manufacturer’s protocol. Briefly, RCC cells of TKT-overexpressed or TKT knockdown or treated with PKM2 siRNA for 24 h were seeded (5 × 10^4^ cells/well) into 96-well plates. Following treatment with different concentration of cisplatin, 10 µl CCK-8 solution was added to each well and incubated for 1 h. The absorbance value of each well at a wavelength of 450 nm was measured using a Cytation3 microplate spectrophotometer (BioTek Instruments, Inc., Winooski, VT, USA). Each experiment was performed in triplicate, and background absorbance was corrected by subtracting the values from blank wells containing medium alone.

### Wound healing assays

For wound healing assays, cells were seeded in 6-well plates and cultured to ~80% confluence. Linear wounds were created using a sterile 10-μl pipette tip. Dislodged cells and debris were removed by washing with PBS, and cells were maintained in serum-reduced medium (1% FBS). Wound closure was monitored at 0 h and 24 h using an inverted phase-contrast microscope (Olympus IX83) at a magnification of 100×. The degree of wound closure was measured at 0 h and 24 h, and the relative migration rate was calculated as follows:

$${\rm{Migration}}\;{\rm{rate}}\;(\%)=[({\rm{Wound}}\;{\rm{width}}\;{\rm{at}}\,0\,{\rm{h}}-{\rm{Wound}}\;{\rm{width}}\;{\rm{at}}\,24\,{\rm{h}})/{\rm{Wound}}\;{\rm{width}}\;{\rm{at}}\,0\,{\rm{h}}]\times 100 \% .$$

### Cell migration and invasion

For migration assays, cells were seeded into the upper chamber of 24-well Transwell plates (10.0 μm pore size; Labselect, Hefei, China). For invasion assays, the upper chamber was pre-coated with Matrigel (356234, Corning, USA). Complete medium with 10% FBS was added to the lower chamber as a chemoattractant. After 24–48 h incubation at 37 °C, non-migrated or non-invaded cells on the upper membrane surface were carefully removed using a cotton swab. Migrated or invaded cells on the lower membrane surface were fixed with 4% PFA, stained with 0.1% Crystal Violet, and imaged under an inverted microscope. Three random fields per membrane were counted, and the percentage of migrated or invaded cells was calculated relative to the total seeded cells.

### Glucose uptake, lactate production and ATP production

For glucose uptake and lactate production detection, transfected 786-O and ACHN cells were seeded in 6-well plates and cultured to ~60% confluence. The culture medium was replaced, and cells were further incubated overnight. The conditioned medium was collected and analyzed for glucose uptake and lactate production using a glucose assay kit (#A154-1-1, Jiancheng Bioengineering Institute, Nanjing, China) and a lactate assay kit (#A019-2-1, Jiancheng Bioengineering Institute, Nanjing, China), respectively, following the manufacturer’s instructions. Cells were harvested and counted using an automated cell counter for normalization in subsequent statistical analyses.

For ATP generation assay, cellular ATP levels were measured using a commercially available ATP assay kit (#S0026, Beyotime Biotechnology, Shanghai, China) according to the manufacturer’s instructions. Briefly, transfected cells were seeded in a 6-well plate at a density of 1 × 10^5^ cells per well and cultured to ~90% confluence. Cells were lysed with the provided lysis buffer, and the lysates were centrifuged at 12,000 × *g* for 5 min at 4 °C to remove debris. The supernatant was mixed with the ATP detection working solution, and luminescence intensity was measured immediately with Cytation3 microplate spectrophotometer (BioTek Instruments, Inc., Winooski, VT, USA). ATP levels were normalized to total protein content, determined by a BCA protein assay, and expressed as relative luminescence units (RLU) per mg of protein.

### Immunohistochemistry (IHC)

IHC assays were conducted following a standard streptavidin-peroxidase method as previously reported [66]. The tissues or TMA slides were dewaxed at 60 °C for 20 min and then washed (3 × 10 min) with xylene followed by rehydrated with graded ethanol and finally distilled water. Endogenous peroxidase activity was quenched with 3% hydrogen peroxide for 10 minutes, and antigen retrieval was conducted using citrate buffer (pH 6.0) with microwave technique. After cooling, slides were blocked with 5% bovine serum albumin (BSA) for 30 min and incubated overnight at 4 °C with primary antibodies. Slides were then incubated with horseradish peroxidase (HRP)-conjugated secondary antibodies for 1 h at room temperature, followed by visualization using 3,3′-diaminobenzidine (DAB) and counterstaining with hematoxylin. Finally, sections were dehydrated, cleared in xylene, and mounted with a coverslip for microscopic analysis.

For primary antibody, anti-TKT antibody was used with 1:400 dilution, anti-Ki-67antibody (12202S, Cell Signaling Technology, Danvers, MA, USA) was used with 1:100 dilution.

### Evaluation of immunostaining

The immunostaining signals were quantified based on both the intensity of staining and the percentage of positively stained cells. The staining intensity of TKT was scored on a scale of 0–3 (0 = negative; 1 = weak; 2 = moderate; 3 = strong), while the percentage of positive cells was categorized into four groups: 1 (0–25%), 2 (26–50%), 3 (51–75%), and 4 (76–100%). The immunohistochemical score (IRS) for TKT staining was calculated by multiplying the intensity score by the percentage score. Based on the IRS, TKT expression levels were classified as low (IRS: 1–4) or high (IRS: 6–12). To ensure objectivity, the evaluation of TKT staining was performed independently and blindly by two experienced pathologists.

### Animal work

Female BALB/c nude mice (6–8 weeks old) were purchased from Beijing Vital River Laboratory Animal Technology Co., Ltd. (Beijing, China). All animal experiments were approved by the Animal Care and Use Committee of Xuzhou Medical University and conducted in accordance with institutional guidelines.

For the subcutaneous tumor model, 2 × 10⁶ ACHN-Ctrl/shTKT/ OE-TKT cells mixed in Corning Matrigel with 1:1 volume ratio was subcutaneously inoculated into the ventral flanks of each mouse (*n* = 6 for each group). Tumor volume was measured weekly using the formula V = *a* × (*b*²)/2, where *a* represents the largest diameter and *b* represents the smallest diameter. The experiment was terminated once the tumor volume exceeded 300 mm³ to ensure compliance with ethical guidelines. Tumors were harvested and weighted at endpoint. IHC was performed to assess the tumor growth of xenograft model.

For the lung metastasis model, luciferase-labeled ACHN-vector and luciferase-labeled ACHN-OE-TKT cells (3 × 10⁶ cells/mouse) were injected into the mice via the caudal vein. Bioluminescence imaging was performed 4 weeks post-injection to assess lung metastasis.

### Statistical analysis

Statistical analyses were performed using GraphPad Prism 9.0 software. The association between TKT expression and clinicopathological parameters in RCC patients was assessed using the Chi-square test. Kaplan–Meier survival analysis with log-rank tests was employed to evaluate the correlation between TKT expression and patient survival. Comparisons among multiple groups were conducted using one-way ANOVA, while differences between control and overexpression groups were analyzed using a two-tailed Student’s *t* test. Data are presented as mean ± standard deviation (SD). *P < 0.05; **P < 0.01; ***P < 0.001；****P < 0.0001. Bars represent means ± SD.

## Supplementary information

Supplemental Figures and Figure legends

Original Data

## Data Availability

The datasets used and/or analyzed during the current study are available from the corresponding author on reasonable request.
