# Supplementary material for: TKT drives renal cell carcinoma progression through metabolic reprogramming and synergistic interaction with PKM2
Source: Cell Death Discov. 2025 Nov 18;11:537. doi: 10.1038/s41420-025-02837-7 (PMC12627471; doi:10.1038/s41420-025-02837-7)
Supplement: Supplementary file 1 — Supplemental Figures and Figure legends [file 41420_2025_2837_MOESM1_ESM.docx]

**
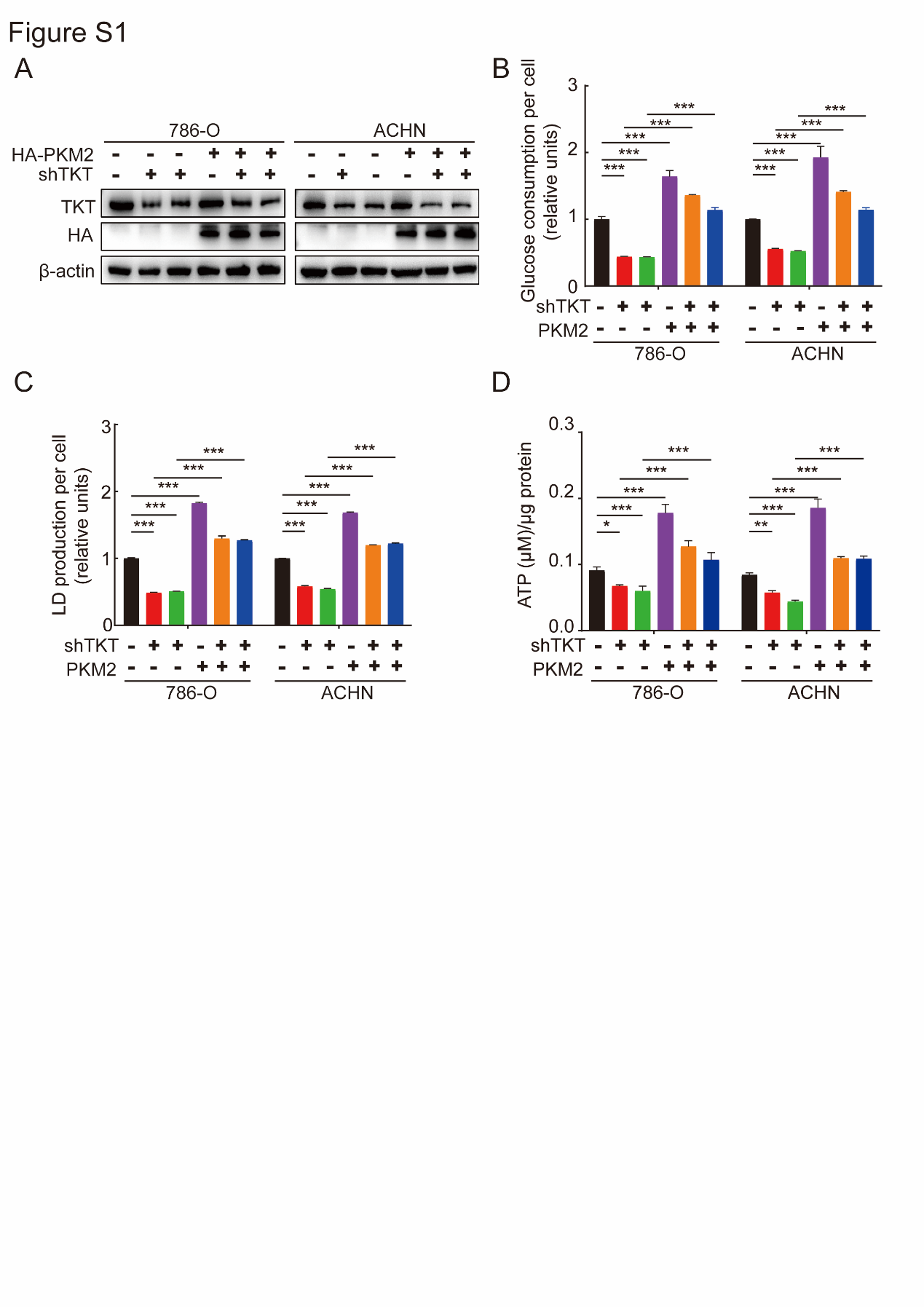
**

**Figure S1. Aerobic glycolysis of PKM2 overexpression in TKT-knockdown RCC cells.** (A) Western blot confirming PKM2 overexpression in control and TKT-knockdown 786‑O/ACHN cells. (B) The glucose uptake of PKM2 overexpression in TKT-knockdown 786‑O/ACHN cells. (C) The lactic acid production of PKM2 overexpression in TKT-knockdown 786‑O/ACHN cells. (D) The ATP production of PKM2 overexpression in TKT-knockdown 786‑O/ACHN cells. *P < 0.05, **P < 0.01, ***P < 0.001*.*


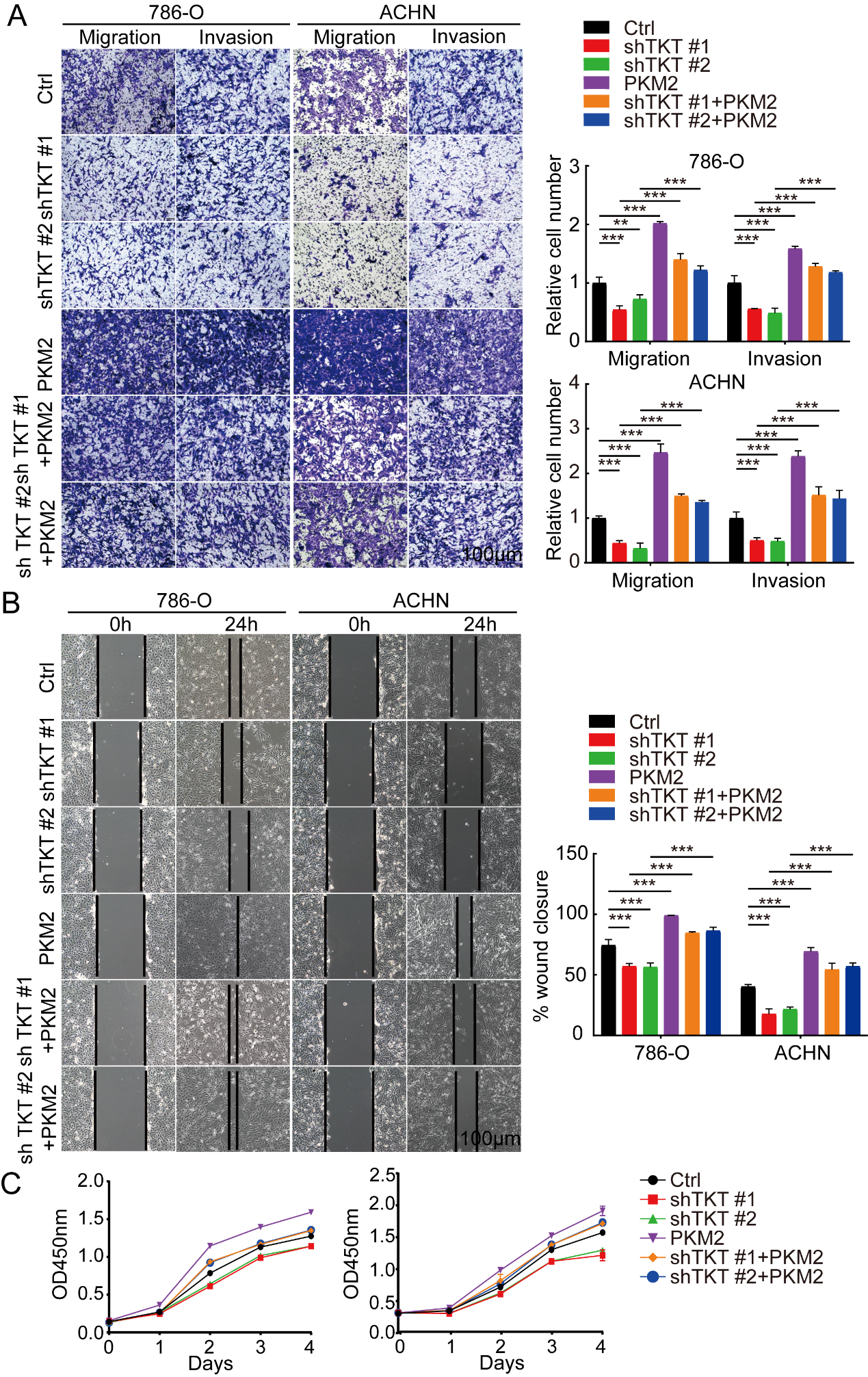


**Figure S2. Metastasis and proliferation of PKM2 overexpression in TKT-knockdown RCC cells.** (A) Transwell assays assessing the impact PKM2 overexpression in TKT-knockdown RCC cells. (B) Wound healing assays demonstrating that PKM2 overexpression reverses TKT knockdown-driven wound closure in RCC cells. (C) CCK-8 assays assessing the impact of PKM2 overexpression on TKT knockdown-driven RCC cell proliferation. Relative statistical results of CCK-8 assays are as following. In 786-O cells, Day 2: Ctrl vs shTKT #1: ***; Ctrl vs shTKT #2: ***; Ctrl vs PKM2: ***; shTKT #1 vs shTKT #1+ PKM2: ***; shTKT #2 vs shTKT #2+ PKM2: ***. Day 3: Ctrl vs shTKT #1: ***; Ctrl vs shTKT #2: ***; Ctrl vs PKM2: ***; shTKT #1 vs shTKT #1+ PKM2: ***; shTKT #2 vs shTKT #2+ PKM2: ***. Day 4: Ctrl vs shTKT #1: ***; Ctrl vs shTKT #2: ***; Ctrl vs PKM2: ***; shTKT #1 vs shTKT #1+ PKM2: ***; shTKT #2 vs shTKT #2+ PKM2: ***. In ACHN cells, Day 2: Ctrl vs shTKT #1: ***; Ctrl vs shTKT #2: **; Ctrl vs PKM2: ***; shTKT #1 vs shTKT #1+ PKM2: ***; shTKT #2 vs shTKT #2+ PKM2: ***. Day 3: Ctrl vs shTKT #1: ***; Ctrl vs shTKT #2: ***; Ctrl vs PKM2: ***; shTKT #1 vs shTKT #1+ PKM2: ***; shTKT #2 vs shTKT #2+ PKM2: ***. Day 4: Ctrl vs shTKT #1: ***; Ctrl vs shTKT #2: ***; Ctrl vs PKM2: ***; shTKT #1 vs shTKT #1+ PKM2: ***; shTKT #2 vs shTKT #2+ PKM2: ***. *P < 0.05, **P < 0.01, ***P < 0.001.
