# Supplementary figures and images for: TKT drives renal cell carcinoma progression through metabolic reprogramming and synergistic interaction with PKM2
Source: Cell Death Discov. 2025 Nov 18;11:537. doi: 10.1038/s41420-025-02837-7 (PMC12627471; doi:10.1038/s41420-025-02837-7)

Figure 2A

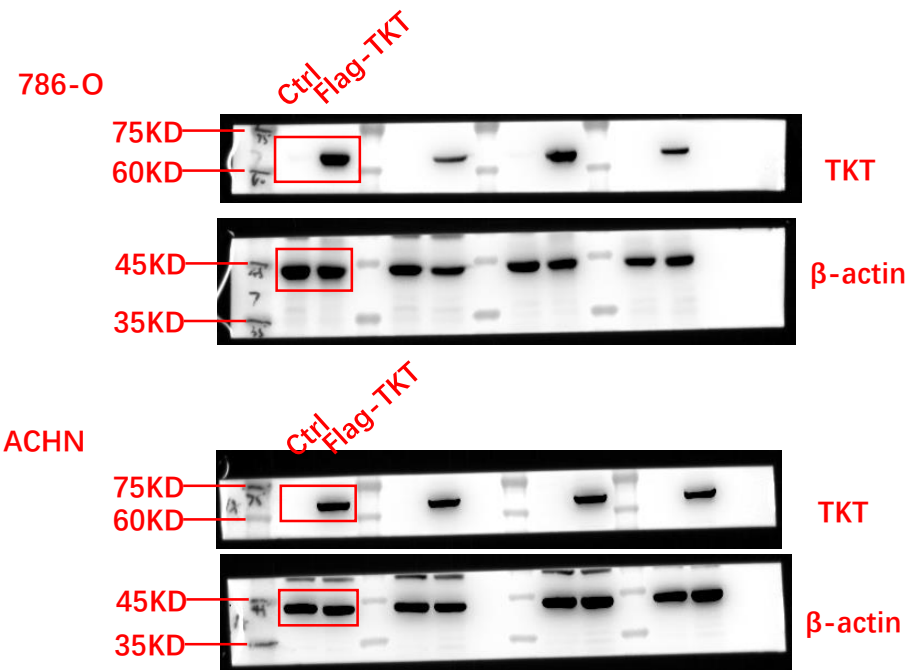

Figure 2C

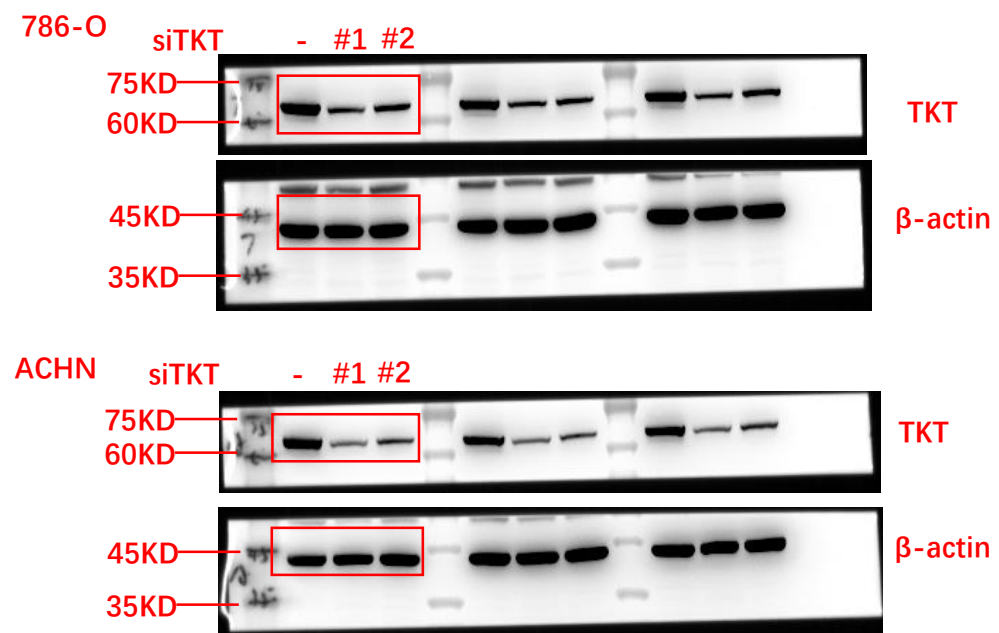

Figure 4C

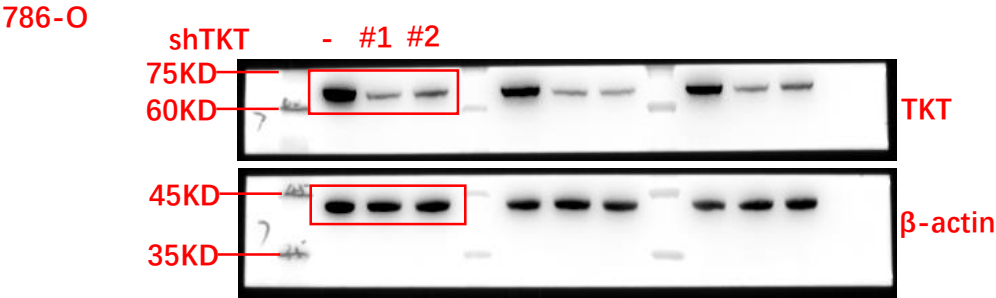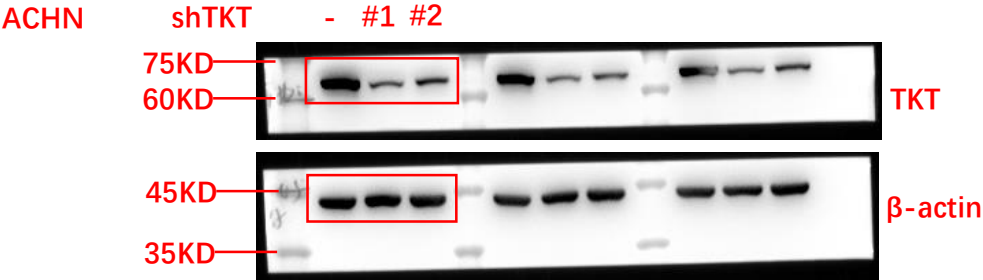

Figure 4H

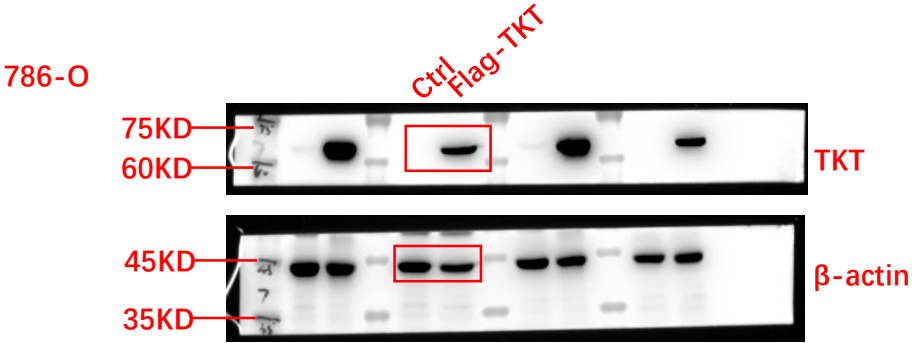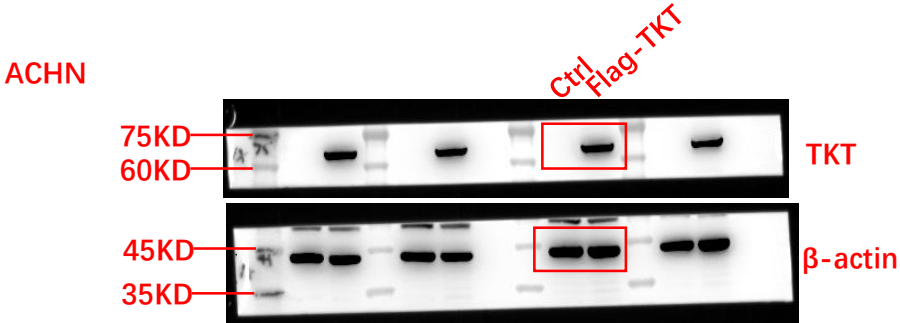

Figure 6B

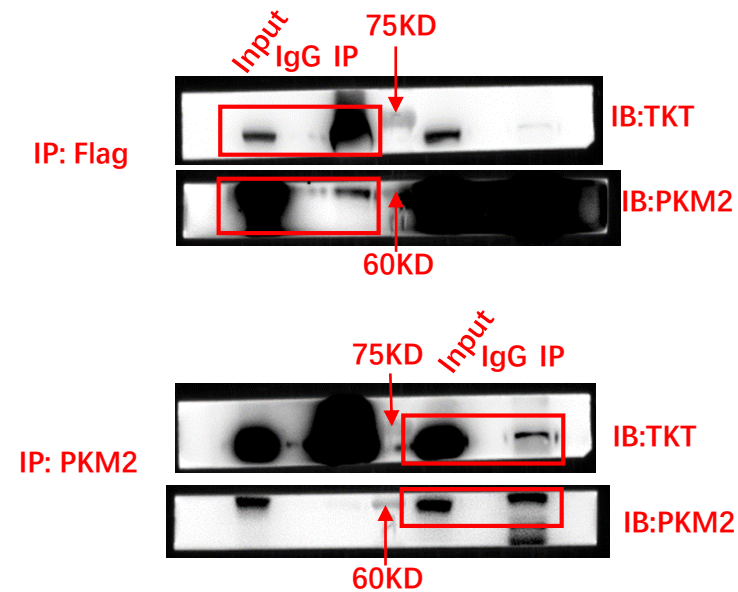

Figure 6C

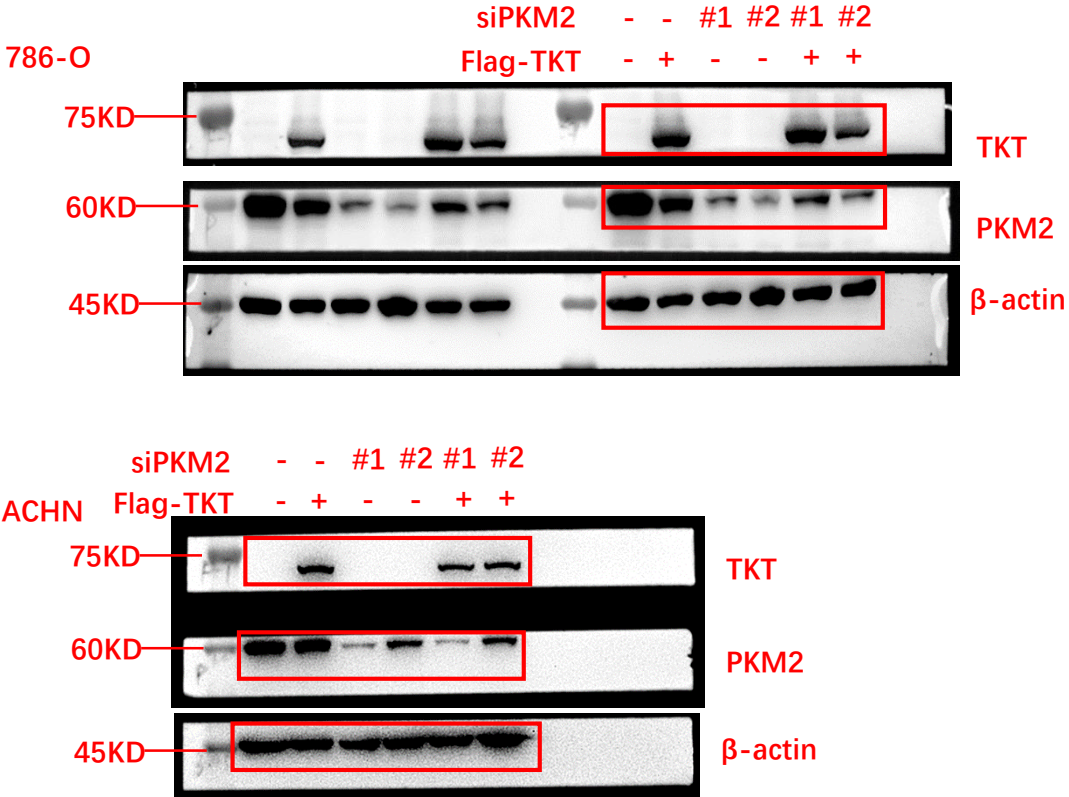

Figure S1A

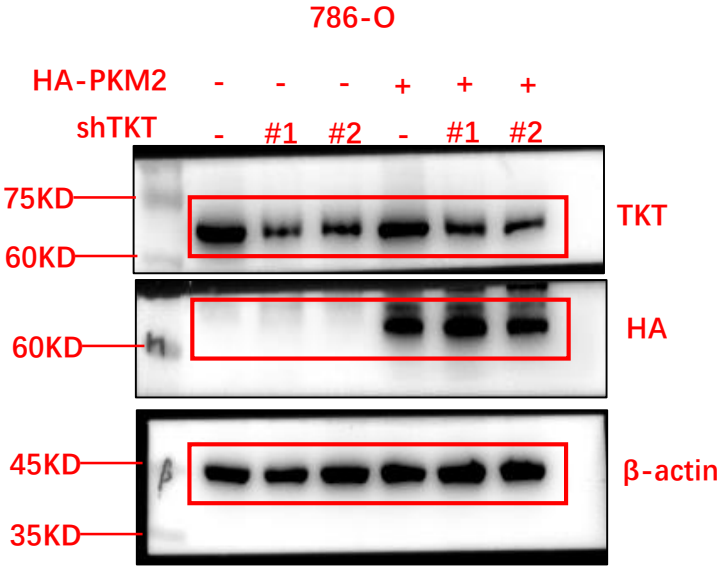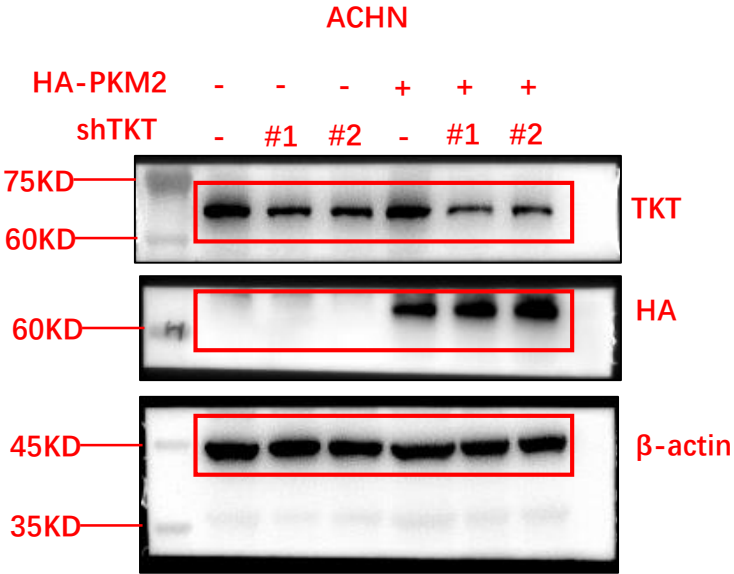

Supplement: Supplementary file 2 — Original Data [file 41420_2025_2837_MOESM2_ESM.pdf]
